# Editorial: Multidrug-resistant Gram-negative bacteria in fragile hosts

**DOI:** 10.3389/fmicb.2026.1865417

**Published:** 2026-05-07

**Authors:** Francesca Montagnani, Andrea Lombardi, Alessandra Mularoni, Jean-Denis Docquier, Francesco Marchesi

**Affiliations:** 1 Department of Medical Biotechnologies, University of Siena, Siena, Italy; 2 Infectious and Tropical Diseases Unit, Department of Medical Sciences, Siena University Hospital, Siena, Italy; 3 Infectious Diseases Unit, Foundation Istituto di Ricovero e Cura a Carattere Scientifico (IRCCS) Ca' Granda Ospedale Maggiore Policlinico, Milan, Italy; 4 Department of Pathophysiology and Transplantation, University of Milan, Milan, Italy; 5 Centre for Multidisciplinary Research in Health Science (MACH), University of Milano, Milan, Italy; 6 Unit of Infectious Diseases, Istituto Mediterraneo per i Trapianti e Terapie ad Alta Specializzazione - Istituto di Ricovero e Cura a Carattere Scientifico (ISMETT-IRCCS), Palermo, Italy; 7 Clinical and Experimental Oncohematology Unit, IRCCS Regina Elena National Cancer Institute, Rome, Italy

**Keywords:** cancer, Gram-negative bacteria, hematological malignancies, immunocompromised patients, multidrug resistant (MDR), transplant

In vulnerable and high-risk individuals, such as solid organ transplant (SOT) or hematopoietic stem cell transplant (HSCT) recipients and solid or hematologic cancer patients, colonization and infection by multidrug-resistant organisms (MDROs), a formidable global health issue *per se*, represent an even more significant threat. These conditions pose meaningful challenges in clinical management, particularly in prevention, diagnosis, and treatment. The main aim of this Research Topic was to focus on Gram-negative MDROs in fragile hosts, with a particular emphasis on carbapenem-resistant Enterobacterales (CRE) and difficult-to-treat non-fermentative Gram-negative bacteria (DTR-NFGN). Within this topic, 13 articles have been published that deepen our understanding of the epidemiology, risk factors, prevention, and therapy of CRE and DTR-NFGN in this vulnerable population.

Among CRE, KPC-type carbapenemase-producing *Klebsiella pneumoniae* (KPC-Kp) isolates are a frequent cause of healthcare-related infections in hospitals or long-term care facilities. Wan et al. characterized ST792 KPC-Kp strains isolated in Jiangxi Province, China. Though rarely documented globally, ST792 has emerged as a concerning threat in southern China. Whole-genome sequencing (WGS) revealed seven ST792 *K. pneumoniae* strains from clinical respiratory specimens, with phylogenetic analysis categorizing them into three subgroups, indicating clonal transmission. Beyond *bla*_KPC − 2_, strains harbored multiple plasmid-encoded resistance genes, facilitating horizontal gene transfer.

The concern regarding in-hospital spreading of KPC-Kp with resistance to new antibiotics was further explored by Nicitra et al.. They analyzed ceftazidime/avibactam-resistant KPC-Kp isolates from immunocompromised patients at an Italian transplant center. WGS revealed ST101 prevalence, potential for hypervirulent and hypermucoviscous phenotypes, and plasmid distribution associated with sequence typing, confirming both clonal spread and horizontal gene transfer as key factors in MDRO dissemination.

Among DTR-NFGN, *Acinetobacter baumannii, Stenotrophomonas maltophilia*, and *Pseudomonas aeruginosa* are the main etiological agents of difficult-to-treat infections in fragile hosts. A less frequent but emerging DTR-NFGN is *Elizabethkingia miricola*. The study by Chen et al. analyzed isolates of *E. miricola* from three patients in the USA, performing a comparative genomic analysis of human and frog isolates. These showed an exceedingly broad resistance to antibiotics, with susceptibility only to trimethoprim/sulfamethoxazole and ciprofloxacin, while intrinsic metallo-β-lactamases and virulence genes were conserved across clinical and frog isolates, highlighting the relevance of the One Health concept.

*A. baumannii*, a well-known cause of difficult-to-treat nosocomial infections, is frequently extensively drug-resistant (XDR) or pandrug-resistant (PDR) due to its ability to acquire or upregulate resistance determinants. Fox et al. characterized four *A. baumannii* strains isolated at different time points from a cystic fibrosis patient initially colonized by mucoid and non-mucoid MDR OXA-23-producing *A. baumannii* isolates. After inhaled tobramycin and colistin treatment, the non-mucoid phenotype was lost, while mucoid strains persisted, with one evolving concomitant resistance to colistin and cefiderocol. WGS revealed that all isolates belonged to ST2, indicating that they evolved from the same strain. Mucoid strains lost virulence traits over time but persisted via biofilm-mediated protection, highlighting the role of antibiotic exposure in XDR/PDR emergence.

Antibiotic exposure was also a key risk factor related to XDR *A. baumannii* infections in the retrospective analysis by Shi et al.. Variables such as ICU length of stay, hypoproteinemia, glucocorticoid use, urinary catheters, mechanical ventilation, diabetes mellitus, and use of carbapenems or β-lactam/β-lactamase inhibitor combinations were all significantly associated with XDR *A. baumannii* infection. A nomogram for predicting XDR *A. baumannii* nosocomial infection risk was proposed, providing a useful model for early identification and mitigating avoidable risks.

Antimicrobial stewardship begins with enhanced attention to prophylactic measures. While the use of fluoroquinolones (FQs) is recommended in high-risk (HR) hematological patients, their role is still debated. In the MDRO era, the wide use of FQs should be limited. In a retrospective study, Santoni *et al*. analyzed infectious outcomes in HR hematological patients with prolonged neutropenia, omitting FQs prophylaxis. Rates of infectious mortality were comparable to settings where FQs prophylaxis is universally used, provided an efficient prompt treatment protocol is adopted.

HSCT recipients further represent a vulnerable population, and bloodstream infections (BSIs) are associated with high mortality in this population. Corcione et al. analyzed the prevalence of rectal MDRO colonization in 279 adult HSCT recipients. Rectal colonization, even if not directly linked to increased mortality, was significantly associated with higher subsequent BSIs, which adversely affected overall survival.

Similarly, Mbatia et al. explored Gram-negative (GN)-BSIs at a pediatric oncology center in Egypt. In children with Acute Myeloid Leukemia, 27.4% developed at least one GN-BSI, 80% of which were caused by MDROs, the burden of which was found to be significantly higher in this cohort. This underlines the need for infection control and pediatric-approved novel antibiotics.

Beyond empiric antibiotic choice, early de-escalation is essential in infection control policies. Bono et al. retrospectively demonstrated the feasibility of a specific de-escalation approach in MDRO-colonized HSCT recipients, once blood cultures remained negative and patients were stable.

To reduce antibiotic use while ensuring adequate empirical therapy, it is vital to optimize diagnosis. Chen and Ma reviewed biomarkers for predicting bacterial and fungal infections in patients with hematologic cancers and the use of metagenomic next-generation sequencing to reveal microorganisms that cannot be detected by conventional testing methods. The integration of multiple biomarkers, clinical symptoms, and local epidemiological data can support clinicians in reaching the best diagnostic and therapeutic decisions.

In cancer patients, the clinical management of infectious complications can be more challenging, due to the complex interplay between microbes and tumors. Emerging evidence indicates that microbial interactions within the tumor microenvironment exacerbate cancer progression. Zhu et al. reviewed microbial-tumor interactions and proposed innovative therapeutic strategies that address both malignancy and associated microbial infections.

A peculiar HR population for MDRO infections is represented by lung transplant (LuTx) recipients, due to direct exposure of the allograft to the environment. Sambo et al. provided a narrative review of the challenges posed by MDR-GN bacteria in LuTx recipients. The authors synthesized current evidence on diagnostic approaches, prophylactic measures, and therapeutic management regarding both colonization and infection. Remaining knowledge gaps in this area are also highlighted, emphasizing the need for further research to optimize prevention and treatment strategies in this high-risk population.

Finally, Duan et al. explored the use of antibiotics in preservation fluid to reduce donor-derived infections by carbapenem-resistant Gram-negative bacteria in SOT. *In vitro* results showed that polymyxin B and colistimethate sodium hold potential for decontamination in clinical practice, bypassing the drug's notorious nephrotoxicity during systemic use.

In conclusion, ongoing research and a comprehensive approach to diagnosis, prophylaxis, therapy, and antimicrobial stewardship are essential to combat the spread of MDROs in fragile hosts and to enable optimal clinical management.

